# Investigation on the Effects of a Pulsed Electric Field (PEF) Continuous System Implemented in an Industrial Olive Oil Plant

**DOI:** 10.3390/foods11182758

**Published:** 2022-09-08

**Authors:** Alessandro Leone, Antonia Tamborrino, Sonia Esposto, Antonio Berardi, Maurizio Servili

**Affiliations:** 1 Department of Agricultural and Environmental Science, University of Bari Aldo Moro, Via Amendola 165/A, 70126 Bari, Italy; 2 Department of the Science of Agriculture, Food and Environment, University of Perugia, Via S. Costanzo, 06126 Perugia, Italy

**Keywords:** olive oil, pulsed electric fields, phenols and volatile content, process efficiency, sensory characteristics

## Abstract

The aim of this study was to investigate how the treatment of olive paste of the *Picholine* variety with pulsed electric fields (PEF) under real operating conditions in a large-scale olive oil extraction plant affects the extractability, chemical composition and sensory profile of the oils. The application of pulsed electric fields (PEF) as a non-thermal food processing technology is interesting for many food extraction processes. The results of this study show that pulsed electric fields can be used as a pretreatment before oil separation to increase the extractability of the process and improve the content of functional components. The application of pulsed electric field (PEF) treatment (2.4 kV/cm, 4 kJ/kg, 6 µs pulse width) to olive paste through a continuous system significantly increased the extractability and total concentration of phenols (especially oleuropein derivatives) compared to the control. In addition, the volatile compounds, α-tocopherol, the fatty acid profile and the main legal quality parameters of extra virgin olive oil (EVOO), including free acidity, peroxide values, extinction indices and sensory analysis, were evaluated. The pulsed electric fields (PEF) treatment did not modify these EVOO quality parameters, neither the α-tocopherol content nor the volatile profile. The sensory properties of EVOO were not affected as well as the PEF treatment showed a similar intensity of fruity and pungent attributes without any off-flavor according to the European Union legal standards. An increase in the bitter taste attribute was observed in the PEF oils. Consequently, this study demonstrates that pulsed electric fields (PEF) processing could be implemented in olive oil processing as pretreatment for improving the efficiency of the process.

## 1. Introduction

Currently, PEF (pulsed electric field) technology is attracting great interest in the olive oil industry. The main advantages of PEF technology over conventional technology are improved extraction yield, reduced processing time and, consequently, lower intensity of conventional extraction parameters (i.e., malaxation temperature and time) and improved olive oil quality. Currently, these technologies are also considered to contribute to a reduction in energy costs and environmental impact. Although the quality of the olive oil depends on many pre- and post-harvest factors [1,2,3,4,5,6,7], over the last twenty years, we have witnessed the need for the latest technological developments in oil processing to improve quality and quantity, but the potential for expansion and growth of the olive processing sector is still great. The process includes cleaning of the olives, crushing to break the cell envelopes of the mesocarp cells and release the oil [8], conditioning by malaxation to facilitate the grouping of the small oil droplets into larger droplets [9,10], and subsequent mechanical extraction by centrifugation [11], which is the most common system for separating the olive oil from the olive paste. PEF is considered a non-thermal food processing technology that has been studied to improve mass transfer processes in the food industry. PEF technology involves the application of an external electric field that can induce a critical electric potential at the cell membrane [12,13]. This leads to electrical breakdown and local structural changes of cell membranes, increasing permeability. Several studies have been published on the disintegration of cell membranes in plant tissues by the application of PEF [14,15,16,17]. In particular, Devkota et al. [14] studied the phenomena on common beans, while Naliyadhara et al. [15] highlighted the importance of PEF treatment for the extraction of various compounds from the food matrix such as juices, edible oils, bioactive compounds and carbohydrates and how the cell membrane mechanism is involved in these outcomes. Shorstkii et al. [16] found more increased porous volume parameters of the cells in sunflower oil pilot production.

The literature on PEF treatment of olive paste has shown that the advantages of PEF application include increased process efficiency and high olive oil quality and yield [18,19,20,21,22]. These results were obtained mainly with laboratory-scale plants [19,22] showing the potential of PEF to increase oil yield from fresh olives. Abenoza et al. [19] investigated the effect of PEF treatment compared to different malaxation times and temperatures. They found an improvement in extraction yield when the olive paste was treated with PEF (2 kV/cm) without malaxation, and no effect on the extraction yield was found when the olive paste was malaxed at 26 °C before PEF treatment [19]. They confirmed the benefits of PEF on olive oil extraction yield using monopolar pulses of 3 μs at electric field strengths of 1 kV/cm (1.47 kJ/kg) and 2 kV/cm (5.22 kJ/kg) and a frequency of 125 Hz. A positive effect on olive oil quality, in terms of improving olive oil sensory quality, was also found. Furthermore, in [19], an electric field strength of 1.8 kV/cm, a pulse width of 15 μs, and a frequency of 300 Hz were used with specific energy inputs ranging from 1.6 to 70.0 kJ/kg, and an increase in extraction yield of up to 18% was observed. Nevertheless, to date, there are few published data on the implementation of a PEF system in an industrial olive oil extraction plant and its impact on oil recovery and final product quality. Puértolas and de Marañón [20] implemented a pilot plant with a fully continuous PEF-assisted extraction system in an industrial olive mill (800 kg h^−1^) and investigated the benefits of PEF technology in the production of high quality olive oil, both to increase the extraction yield and to increase the bioactive compound content. The olive pulp was treated with a field strength of 2 kV/cm and 65 J per pulse at a frequency of 25 Hz, resulting in a specific energy of 11.25 kJ/kg. They found that PEF improved the yield of virgin and extra virgin olive oil and increased oil production and the content of health-related substances such as polyphenols, phytosterols and tocopherols. Another study by Tamborrino et al. [23] using an industrial scale pilot plant operating at a mass throughput of 2300 kg/h and a specific energy input of 7.83 kJ/kg showed that PEF is a useful tool to improve process efficiency, both from a quantitative and qualitative point of view. In conclusion, the incorporation of PEF treatment in olive oil processing is still new, and few data are available from industrial-scale plants. However, in order to introduce PEF technology in olive oil mills, pilot-scale extraction studies need to be carried out to confirm previous results, using several varieties and testing different PEF parameters. Therefore, this study investigated the impact of PEF technology in a continuous system in an average olive oil mill on a *Picholine* variety and using a level of specific energy different from those already used in the olive oil extraction process. In particular, the application of pulsed electric fields on oil recovery, general quality parameters, functional food ingredients such as polyphenols, volatile compounds, α-tocopherol, the fatty acid profile and sensory properties of olive oil were investigated. Overall, this work focuses on evaluating the applicability of PEF as a pretreatment in an olive oil extraction mill.

## 2. Materials and Methods

### 2.1. Olive Fruits

Olive fruits of the *Picholine* variety (*Olea europaea* L.) with a maturity index of 2.9 were harvested mechanically by using a trunk shaker and transported to the industrial mill the same day. The degree of ripeness of the fruits was determined according to the method proposed by Uceda and Frias [24]. The olives were produced in Puglia (Lardagnano, SP21, 72017 Ostuni BR; 40.746209, 17.623760) from 30-year-old plants. The production area is flat. The climate of the province is Mediterranean, with hot summers and not excessively cold winters. The rainfall is not abundant and is around 600 mm of rain per year. The olive grove is fertilized and irrigated with a drip system and grown organically. Fertilization is provided for the administration of potassium and phosphorus in autumn with a part of nitrogen at the end of winter and a part in mid-spring. The composition of the soil is mainly characterized by iron and aluminum hydroxides, clay minerals, and quartz components.

### 2.2. Industrial Olive Oil Extraction Plant

**Figure 1:**
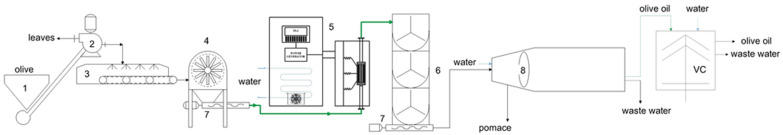
Layout of the plant with the integrated PEF equipment. 1: loading hopper; 2: defoliator; 3: washing machine; 4: crusher machines; 5: continuous PEF system; 6: malaxer machines; 7: cavity pump stators; 8: solid/liquid horizontal centrifugal decanter.

The experimental tests were performed in an industrial olive oil extraction plant (Masseria Asciano s.r.l. Ostuni, BR, Italy). The mill consisted of a set of units built by Rapanelli Fioravante S.p.A., Foligno (PG, Italy): a group of leaf removal and washing machines (model Rapanelli Lasvolea), a knife crusher (model Rapanelli Gr. Inox), a malaxation group with three stacked malaxers with a capacity of 700 kg (model Rapanelli Novoil export), a horizontal three-phase centrifugal decanter for solids and liquids (model Rapanelli Ramef), and a vertical centrifuge (model Rapanelli 4750 eco). The extraction plant has a capacity of 1500 kg/h. The PEF system was located between the crusher and malaxer groups (Figure 1). During the experiments, the PEF system was switched on or off depending on the experimental plan.

### 2.3. Continuous Pilot Scale PEF Assistant System

The PEF treatments were carried out with the continuous system PEF Advantage P 1e (Elea GmbH, Quakenbrück, Germany). The system has the maximum voltage of 24 kV and delivers square wave pulses through a collinear cell with 50 mm gap between electrodes. By generating square wave pulses, *W_pulse* of the PEF system was expressed as a function of the set charging voltage *U*, the current intensity *I* (A) and pulse width *τ* (µs) (Equation (1)):

(1) $$W_{pulse}=U\times 1000\times I\times \tau \;\;\;\;\;\;\;\;\;\;\;\;\;\;\;\;\;\;\;\;\;\;\;\;\;$$

where:

- *W_pulse_* is the energy intensity of a pulse (kJ);
- *U* is the charging voltage (V);
- *I* is the current intensity (A);
- *τ* is the pulse width (µs).

### 2.4. Equipment Set-Up and Trial Planning for PEF Treatment 

The PEF unit was installed in the existing plant between the crusher and the malaxer. Two different arrangements were compared with and without the use of the PEF unit. For each condition test, five trials were performed on homogeneous batches of olives. The sequence of treatments was performed by alternating two control runs (Control) and two PEF runs (PEF). When switching to the next treatment, a cleaning run was performed to replace the treated or untreated paste remaining in the lines. The process parameters used in all tests are listed below:

- the malaxation time was 30 min;
- the malaxation temperature was set at 26 ± 1 °C;
- the mass throughput of the plant was 1.5 t/h;
- the addition of process water to the decanter was about 25%.

In this series of experiments (PEF), the field strength was set to 2.4 kV/cm and the pulse width to 6 µs. During continuous processing of the medium, *W*_*specific* was set to 4 kJ/kg and calculated by the system according to Equation (2):

(2) $$W_{specific}=\frac{W_{pulse}\times f_{pulse}}{m}$$

where:

- *W*_*specific* is the specific energy intensity of the treatment (kJ/kg);
- *W*_*pulse* is the energy intensity of a pulse (kJ);
- *f*_*pulse* is the pulse frequency (Hz);
- *m* is the mass flow rate of the system (kg/s).

### 2.5. Sampling

In each test, the incoming olives, pomace and effluent exiting the decanter, and finished oil were sampled to determine the extractability of the olive oil, oil loss in the pomace and effluent, and olive oil quality parameters. The pomace was removed from the decanter at regular intervals during each test run.

### 2.6. Moisture and Oil Content of Olives and Pomace

Moisture content (% *w*/*w*) was calculated after drying the milled olive and pomace from the decanter at 105 °C to constant weight. The total oil content of the dried milled olive and pomace was determined according to the analytical method described in Squeo et al. [25].

### 2.7. Extractability

The extraction parameters of the oil contained in the pomace and wastewater were used to evaluate the quantitative performance of the oil extraction plant. The oil extraction capacity (*E*) is the ratio between the percentage of oil extracted from the olives by the plant (*Oe*) and the percentage of oil content in the olives (*Oo*). *E* was calculated using the following equation:

(3) $$E=\frac{Oe}{Oo\;}\times 100$$

### 2.8. Analysis of Olive Oil Quality

#### 2.8.1. Legal Quality Indices

The fatty acid composition, free acidity (FA), peroxide values (PV), and extinction indices (K_232_, K_270_ and ΔK) of all EVOO samples were evaluated as required by European Union regulations [26]. 

#### 2.8.2. Phenol Compounds

The hydrophilic phenolic compounds of EVOO were extracted following the method described by Selvaggini et al. [27] with some modifications reported by Taticchi et al. [28] and analyzed by HPLC equipped with diode array and fluorescence detectors (HPLC-DAD-FLD; Agilent Technologies, Santa Clara, CA, USA). 

The α-Tocopherol content was evaluated with the same equipment as mentioned above and as reported by Veneziani et al. [29]. 

#### 2.8.3. Volatile Compounds

The volatile compounds were determined using headspace solid-phase micro-extraction (HS-SPME) followed by gas chromatography/mass spectrometry (HS-SPME-GC/MS; Agilent Technologies, Santa Clara, CA, USA) as described by Taticchi et al. [28].

#### 2.8.4. Sensory Analysis

The procedure for assessing the organoleptic characteristics of EVOO was carried out by using the method reported in standard protocol of IOC [30].

### 2.9. Data Processing

The MATLAB^®^ machine learning and statistical toolboxes were used for the experimental data processing. The significance among means of group of data was detected by the *t* test (*p* < 0.05).

## 3. Results and Discussion

### 3.1. Impact of PEF Treatment on Extractability

Table 1 shows the quantitative results detected in the by-products of the olive oil extraction process. The pomace samples treated with PEF technology have a significantly lower content of residual olive oil than untreated samples. This demonstrates the ability of PEF treatment to improve the extraction of olive oil from the matrix with a significant percentage increase of more than 3% 

Recently, pulsed electric field (PEF) has been used to enhance oil extraction in various matrices. The principle of PEF is to disintegrate the cell membrane structure to increase extraction by applying an electric field. PEF can increase mass transfer during extraction by electroporating the membrane structure of plant materials to improve extraction and shorten extraction time [31]. Published studies confirm that PEF can also be applied to plant materials as a pretreatment method before conventional extraction to shorten extraction time [13,32]. The results found in the current study are comparable to the improvements obtained by other authors in previous studies [19,20,22,23].

### 3.2. Impact of PEF on General Chemical Parameters of Extra Virgin Olive Oil

The main regulatory quality parameters, including FA, PV, K_232_, K_270_ and ΔK, measured for the control and PEF-treated EVOO samples, are listed in Table 2. Both EVOOs were found to have values below the legal limits for the extra virgin category set in the current EU regulation [26], with no significant differences (*p* > 0.05) between them. These results are in agreement with those of previous works [20,29] and confirm that the FA, PVs and extinction indices were not affected by the PEF treatment of the olive paste.

Fatty acid composition plays an important role in the oxidative stability, nutritional value and health value of EVOO. Extensive data from the scientific literature has shown that low levels of saturated fatty acids (FSA) and high levels of monounsaturated fatty acids (MUFA), especially oleic acid, reduce the risk of cardiovascular and atherosclerotic diseases and protect against various cancers [33]. In addition to the health benefits, the high content of MUFA and the low concentration of polyunsaturated fatty acids (PUFA) are key factors for the stability of EVOO. Table 3 shows the fatty acid composition of the control and PEF oils obtained from olive paste of the *Picholine* variety. In both EVOOs, MUFA accounted for more than 74% of the total fatty acid content, followed by SFA and PUFA, which reached 15% and 10%, respectively. As expected, oleic acid was the most abundant (75%), while palmitic and linoleic acids were found in lower concentrations (12% and 9%, respectively). The results show that the PEF treatment had no significant effect on the content of SFA, MUFA and PUFA in EVOO. A similar result was found for oleic acid (see Table 3). These results are in agreement with those reported in some other available studies on the effect of PEF on the fatty acid composition of EVOO, which is strongly correlated with genetic, agronomic and pedoclimatic variables [19]. Abenoza et al. [19] evaluated the effects on the quality parameters of oil from *Arbequina* olive paste treated with PEF before the malaxation phase and concluded that there were no significant differences in SFA, MUFA and PUFA content between PEF-treated and control oil samples. 

### 3.3. Impact of PEF on Phenolic Content and of Extra Virgin Olive Oil

In recent decades, the influence of phenolic compounds of EVOO on biological and sensory properties has been widely demonstrated [33]. These compounds (especially oleuropein derivatives) exhibit strong antioxidant activity by reacting through several mechanisms. For this reason, they are extremely important for maintaining a long shelf life and promoting health benefits [34], which is confirmed by the EFSA health claim [35]. The qualitative and quantitative phenolic composition of EVOO depends on genetic and geographical origin, agronomic and technological factors [33,36] and storage conditions [34]. The phenolic profile of the control EVOO and the PEF-EVOO samples is shown in Table 4. As can be seen, the PEF treatment of the olive pastes of the *Picholine* variety has a significant influence (*p* < 0.05) on the total content of hydrophilic phenols in the EVOO. The PEF-EVOO is characterized by a higher total phenolic content (644.4 ± 27.6 mg kg^−1^) than the EVOO obtained by the traditional mechanical extraction process (583.6 ± 16.8 mg kg^−1^). Specifically, the most significant differences were measured for the oleuropein derivatives, with the content of 3.4 DHPEA-EDA in particular reaching a significant (*p* < 0.05) increase of up to 16.1% with the PEF treatment, with 57.5% for the control EVOO and 60.4% for the PEF-EVOO. In addition, the content of ligstroside derivatives and lignans in the PEF-EVOO sample was slightly higher than in the control EVOO sample, although their concentration did not vary significantly. These results could be explained by the phenomenon of electroporation that occurs during PEF treatment. It promoted the migration of intracellular water, spreading solutes into the external medium, resulting in increased mass transfer from the cell pores into the solution, while enhancing the recovery of target substances [37]. This seems to promote the release and transfer of polar molecules such as 3,4- DHPEA-EDA into the oil phase, which is known to slow down the negative oxidative phenomena in EVOO [34] and improve sensory and health properties [33]. The higher amount of oleuropein derivatives in EVOOs obtained after PEF treatment in olive pastes observed in this work is consistent with previous studies [19,23,29,38]. The ability of PEF technology to improve the solubility of oleuropein derivatives has already been described by Veneziani et al. [29], who observed an increase in these phenolic compounds responsible for health-promoting benefits depending on the different olive varieties and their maturity index. 

In contrast to the increase in hydrophilic phenolic compounds (especially oleuropein derivatives), there were not remarkable differences between PEF and control EVOOs in the concentration of α-tocopherol, the main lipophilic natural antioxidant, which similar to vitamin E, has potent biological activity (Table 4). The α-tocopherol content was 318.4 ± 2.8 and 322.7 ± 3.9 mg kg^−1^ for the control EVOO and PEF-EVOO samples, respectively. These results could be explained by the lipophilicity of α-tocopherol, which promotes its solubility in the oil phase. However, previous studies suggest that the variability of the content of this compound in EVOO is not influenced by processing conditions, while its actual content in oils depends on genetic and agronomic factors [39]. Our results are supported by a recent work [38] that investigated the differential effects of PEF treatment on secoiridoid derivatives as well as on tocopherol content. Our results differ from those of Abenoza et al. [19] and Puértolas et al. [20], who reported a higher increase in α-tocopherol content in EVOO from PEF treatment compared to EVOO from the traditional mechanical extraction process. 

### 3.4. Impact of PEF on Volatile Compounds of Extra Virgin Olive Oil 

The SPME-GC /MS analysis of the two EVOO head ranges is shown in Table 5. Apart from the C5 and C8 ketones, the volatile profile of both EVOOs consisted mainly of C5 and C6 aldehydes, alcohols and esters originating from the lipoxygenase pathway (LOX) and responsible for the “green” and “fruity” sensory notes of the EVOOs [39,40,41]. These compounds did not show significant (*p* > 0.05) differences between control EVOOs and PEF-EVOO. In particular, the C5 and C6 aldehydes were the main volatile compounds shaping the profile of both EVOOs, accounting for 60% of the total content. This fraction was almost exclusively represented by (E)-2-hexenal (56% of the total volatile compounds). Furthermore, lower amounts of alcohols and esters were found in both EVOOs (28% and 10%, respectively). The results show that the PEF treatment had no effect on the volatile content in EVOO. This new technology does not imply any change in the enzymatic activity of the individual enzymes of the LOX pathway. Our results are consistent with previous studies comparing the health and sensory quality of EVOOs from traditional mechanical extraction and PEF-treated EVOOs [29].

### 3.5. Impact of PEF on Sensory Evaluation of Extra Virgin Olive Oil 

In order to better study the impact of the continuous PEF system for the treatment of olive paste on an industrial scale on the organoleptic properties of EVOO, the sensory evaluation was carried out according to the IOC method [30]. Table 6 shows the sensory profile of the EVOOs. Both oils analyzed by the experts belonged to the marketable category “extra virgin”, which according to the current regulations [26,30] is characterized by a median of defects of zero or less (=0) and a median of fruitiness of more than zero (>0). These results indicate that the PEF technology does not impart an off-flavor to the oil, as has already been observed by several other authors [19,20,38] in oils of the *Arbequina*, *Arroniz* and *Manzanilla* varieties. As shown in Table 6, both the control oils and the oils obtained by treating the olive pastes with PEF had a higher “bitter” aftertaste than the control oils, which is related to the increase in phenolic concentration in the oils obtained with PEF technology. Finally, the influence of PEF technology on the “fruity” attribute seems to be similar between the control group oils and the PEF oils, confirming the results in Table 5, which show no significant differences between the control group oils and the PEF oils for the volatile compounds associated with the positive sensory attributes of virgin olive oil.

## 4. Conclusions

The present study on the use of PEF technology for the treatment of olive paste on an industrial scale provided promising results. The results underlined the potential of PEF technology to improve the recovery of bioactive compounds. This technology can be used as a pretreatment for conventional extraction processes and is applicable on an industrial scale. Existing oil mills can be easily retrofitted by installing a PEF unit with a simple flange coupling in the olive paste transfer line from the oil mill to the malaxers. The system needs to be supplied with electricity and cooling water; with an energy input of 4 kJ/kg, the energy requirement corresponds to 1.1 kWh/t of paste to be processed and a temperature increase of ~1 °C. The system can be conveniently placed in the processing area in line with the other plant equipment. The increasing extraction capability of the process justifies the energy requirement of this technology as a new method for recovering oil in the olive paste matrix with positive effects on the quality of the final products. The similar values of fatty acid composition, FA, PVs and spectrophotometric indices found for the different EVOOs indicate that the PEF treatment did not negatively affect the marketable quality of the EVOOs. PEF-EVOO had a higher content of phenolic compounds and oleuropein derivatives (especially 3,4- DHPEA-EDA) compared to the control EVOO, while the content of other phenolic compounds such as secoiridoid derivatives (especially ligstroside), lignans and α-tocopherol did not show significant differences.

This highlights that PEF treatment significantly enhanced the transfer of phenolic compounds to the oil phase, and this was particularly pronounced for molecules with high polarity such as 3,4 DHPEA-EDA, which are responsible for antioxidant properties, the sensory note of bitterness, and various healthy properties. The PEF technology did not transfer sensory deficiencies to the oils, but on the contrary, increased the score for the attribute “bitterness”. Furthermore, the activity levels of the individual enzymes involved in the LOX pathway for the production of volatile EVOO compounds, to which positive olfactory properties are attributed, were not inhibited by the PEF treatment.

## Figures and Tables

**Table 1:** Quantitative results and process parameters.

Test Conditions	Pomace		Extractability
	Moisture (%)	Oil (% db)	(%)
Control	61.64 ± 1.91 a	9.92 ± 0.90 a	86.01 ± 1.40 b
PEF	61.02 ± 0.63 a	7.49 ± 1.52 b	89.19 ± 1.15 a

Different letters in columns denote significant statistical differences at *p* < 0.05.

**Table 2:** Legal quality parameters of EVOOs according to EU regulation [26].

Test Conditions	Free Acidity (%)	Peroxide Value (meq O_2_ kg^−1^)	K_232_	K_270_	ΔK
Legal Limits for EVOO	≤0.8	≤20	≤2.50	≤0.22	≤0.01
Control	0.24 ± 0.02 a	5.68 ± 0.93 a	1.562 ± 0.09 a	0.137 ± 0.01 a	−0.003 ± 0.001 a
PEF	0.25 ± 0.01 a	5.85 ± 0.72 a	1.608 ± 0.06 a	0.143 ± 0.01 a	−0.003 ± 0.001 a

Different letters in columns, for each test conditions, denote significant statistical differences among means, *p* < 0.05.

**Table 3:** Fatty acid composition (%) of EVOOs.

Fatty Acid Composition	Control			PEF		
Myristic (C14:0)	n.d			n.d		
Palmitic (C16:0)	12.27	±	0.72 a	12.33	±	0.35 a
Palmitoleic (C16:1)	0.70	±	0.06 a	0.73	±	0.05 a
Margaric (C17:0)	0.06	±	0.004 a	0.06	±	0.01 a
Margaroleic (C17:1)	0.09	±	0.003 a	0.09	±	0.01 a
Stearic (C18:0)	2.38	±	0.05 a	2.37	±	0.23 a
Oleic (C18:1 ω-9)	73.73	±	0.64 a	73.57	±	0.67 a
Linoleic (C18:2 ω-6)	9.22	±	0.20 a	9.33	±	0.40 a
Linolenic (C18:3 ω-3)	0.88	±	0.03 a	0.89	±	0.01 a
Arachidic (C20:0)	0.37	±	0.03 a	0.35	±	0.03 a
Eicosenoic (C20:1)	0.30	±	0.02 a	0.28	±	0.02 a
Behenic (C22:0)	n.d			n.d		
Lignoceric (C24:0)	n.d			n.d		
SFA	15.08	±	0.72 a	15.11	±	0.42 a
MUFA	74.82	±	0.64 a	74.67	±	0.67 a
PUFA	10.10	±	0.20 a	10.22	±	0.40 a

Different letters in rows, for each test conditions, denote significant statistical differences among means, *p* < 0.05.

**Table 4:** Phenolic composition of EVOOs. Data expressed as mg kg^−^^1^.

Phenols	Control	PEF
3,4-DHPEAa	5.5 ± 1.1 a	7.8 ± 1.7 a
*p-*HPEA	6.3 ± 1.3 a	6.7 ± 0.3 a
Vanillic acid	0.3 ± 0.1 a	0.3 ± 0.0 a
3,4-DHPEA-EDA	335.4 ± 11.2 b	389.5 ± 22.2 a
*p*-HPEA-EDA	75.9 ± 5.3 a	76.8 ± 4.6 a
(+)-1-acetoxypinoresinol	12.8 ± 1.3 a	13.4 ± 0.4 a
(+)-pinoresinol	9.1 ± 0.3 a	8.0 ± 1.3 a
3,4-DHPEA-EA	124.7 ± 7.7 a	128.7 ± 11.0 a
Ligstroside aglycone	13.5 ± 1.9 a	13.2 ± 1.0 a
Total hydrophilic phenols	583.6 ± 16.8 b	644.4 ± 27.6 a
Oleuropein derivatives	465.6 ± 13.7 b	526.0 ± 24.8 a
Ligstroside derivatives	95.8 ± 5.7 a	96.7 ± 4.7 a
Lignans	21.9 ± 1.4 a	21.4 ± 1.3 a
α-Tocopherol	318.4 ± 2.8 a	322.7 ± 3.9 a

Different letters in rows denote significant statistical differences at *p* < 0.05.

**Table 5:** Volatile compounds detected in EVOOs. Data expressed as µg kg^−1^.

Volatile Compounds	Control	PEF
**Aldehydes**		
Pentanal	67 ± 8 a	65 ± 7 a
(E)-2-Pentenal	36 ± 3 a	32 ± 5 a
Hexanal	556 ± 42 a	553 ± 32 a
(E)-2-Hexenal	10407 ± 634 a	10447 ± 817 a
(E,E)-2,4-Hexadienal	72 ± 7 a	60 ± 4 b
**Sum of C_5_–C_6_ aldehydes**	11,138 ± 635 a	11,157 ± 818 a
**Alcohols**		
1-Pentanol	73 ± 24 a	90 ± 17 a
1-Penten-3-ol	207 ± 14 a	206 ± 23 a
(E)-2-Penten-1-ol	33 ± 1 a	29 ± 1 b
(Z)-2-Penten-1-ol	247 ± 24 a	250 ± 13 a
1-Hexanol	1499 ± 74 a	1511 ±86 a
(E)-2-Hexen-1-ol	1425 ± 141 a	1422 ±123 a
(Z)-3-Hexen-1-ol	1719 ± 168 a	1717 ± 97 a
**Sum of C_5_–C_6_ alcohols**	5203 ± 234 a	5224 ± 182 a
**Esters**		
Hexyl acetate	243 ± 19 a	245 ± 21 a
(Z)-3-Hexenyl acetate	1619 ± 142 a	1621 ± 100 a
**Sum of C_6_ esters**	1861 ± 143 a	1866 ± 102 a
**Ketones**		
3-Pentanone	215 ± 13 a	230 ± 14 a
1-Penten-3-one	141 ± 12 a	112 ± 9 b
6-Methyl-5-hepten-2-one	20 ± 3 a	21 ± 3 a
**Sum of C_5_–C_8_ ketones**	376 ± 18 a	363 ± 17 a

Different letters in rows denote significant statistical differences at *p* < 0.05.

**Table 6:** Median of the sensory attributes of EVOOs.

Sensory Attributes	Control	PEF
Median defects	0.0	0.0
Median fruity	3.1	3.1
Median bitter	3.2	3.9
Median pungent	3.1	3.3

## Data Availability

Data are contained within the article.
